# Pro-inflammatory cytokine ratios determine the clinical course of febrile neutropenia in children receiving chemotherapy

**DOI:** 10.1186/s40348-020-00097-2

**Published:** 2020-06-09

**Authors:** Mira Siegmund, Julia Pagel, Tasja Scholz, Jan Rupp, Christoph Härtel, Melchior Lauten

**Affiliations:** 1 Department of Pediatrics, Pediatric Hematology and Oncology, University of Lübeck, 23538 Lübeck, Germany; 2 Department of Infectious Diseases and Microbiology, University of Lübeck, Lübeck, Germany; 3 German Center for Infection Research (DZIF), partner site Hamburg-Lübeck-Borstel-Riems, Lübeck, Germany

**Keywords:** Febrile neutropenia, Childhood cancer, Cytokines, Biomarker

## Abstract

**Background:**

Febrile neutropenia is a common and serious complication during treatment of childhood cancer. Empirical broad-spectrum antibiotics are usually administered until neutrophil cell count recovery. It was the aim of this study to investigate cytokine profiles as potential biomarkers using in-vitro sepsis models to differentiate between distinct clinical courses of febrile neutropenia (FN).

**Methods:**

We conducted an observational study in FN episodes of pediatric oncology patients. Courses of neutropenia were defined as severe in case of proven blood stream infection or clinical evidence of complicated infection. We collected blood samples at various time points from the onset of FN and stimulated ex vivo with lipopolysaccharide (LPS) and *Staphylococcus epidermidis* (SE) for 24 h. Twenty-seven cytokine levels were measured in the whole blood culture supernatants by a multiplex immunoassay system.

**Results:**

Forty-seven FN episodes from 33 children were investigated. IL-8, IL-1β, and MCP-1 expression increased significantly over time. IL-8, MIP-1α, MIP-1β, MCP-1, and TNF-α showed significantly lower concentration in patients with a clinically severe course of the FN.

**Conclusions:**

Distinct patterns of cytokine profiles seem to be able to determine infectious FN and to predict the severity of its clinical course. If these data can be verified in a multi-centre setting, this may finally lead to an individualized treatment strategy facilitating antibiotic stewardship in these patients.

## Background

Infections are a potentially life-threatening complication during childhood cancer treatment [1]. The empiric use of broad-spectrum antibiotics in patients with febrile neutropenia leads to a significant decrease in mortality and morbidity [2–5]. However, routine use of antibiotics may cause overtreatment of patients developing fever due to non-bacterial reasons, e.g., due to virus infection, mucosal damage, adrenal insufficiency, or chemotherapy itself [6]. In addition, the excessive use of broad-spectrum antibiotics is associated with the emergence of multidrug-resistant organisms, prolonged hospitalization, and structural changes of the gut microbiota [7–10]. Hence, there is an urgent need to evaluate biomarkers to guide individualized treatment strategies for patients at high risk for bacterial infection. Unfortunately, there is a paucity of markers allowing differentiation between infectious and non-infectious as well as bacterial and non-bacterial fever during neutropenia. Markers like C-reactive protein (CRP) and procalcitonin (PCT) are often considered to indicate infections and they are discussed as biomarkers in FN. Current studies suggests that PCT has better discriminatory ability than CRP [11, 12]. In addition, international risk models for the assessment of severity of infection are scarce [13–15] and neither the common infection parameter CRP nor the highly sensitive PCT can reliably differentiate between neutropenic infections and chemotherapy-induced fever [16–18]. The role of cytokines in the onset of inflammation is well known. Nevertheless, their role for the diagnosis and course of bacterial infection has been controversially discussed [12, 16, 18–21].

Within the last years, multiplex arrays for small blood volumes have been established allowing simultaneous measurement of multiple cytokines [22]. In the setting of FN, multiplex arrays are suitable to quantify even small amounts of cytokines [23]. This study aimed at defining cytokine patterns being capable of distinguishing between severe and mild courses of febrile neutropenia in pediatric oncology patients within the first days of a febrile episode. We chose a sepsis ex-vivo model to achieve a clear discrimination between cytokines due to higher cytokine levels. In addition, an ex-vivo model allowed to reduce potential influence factors.

## Methods

### Study cohort

This single-center convenience sample study was conducted at the Department of Pediatric Oncology and Hematology at the University Hospital Schleswig-Holstein, Campus Lübeck. We used blood samples from patients receiving chemotherapy and developing episodes of febrile neutropenia between 2005 and 2012. Samples were collected at days 0, 1, 4, 7, 10, and 14 of the episodes. Day 0 was defined as the time directly before administration of the antibiotics. Samples of 53 patients were collected and samples of 33 children with 47 febrile episodes were included into our study. We excluded children younger than 18 months, children after allogeneic stem cell transplantation, children with progressive oncological disease or underlying genetic syndrome, and patients with absent clinical data or blood samples (a minimum of three blood samples including day 1, 4, and day > 4 were required). We included a maximum of three episodes of each patient. The cohort was divided into two groups: hematological malignancies (acute lymphoblastic leukemia, acute myelogenous leukemia, Hodgkin’s lymphoma, Non-Hodgkin lymphoma, Langerhans cell histiocytosis) and solid malignancies (Ewing sarcoma, rhabdomyosarcoma, medulloblastoma, neuroendocrine ovarian carcinoma, desmoplastic small round cell tumor, low-grade astrocytoma). Patients with FN were analyzed in two risk groups according to clinical and microbiological findings: The first group (severe course group, SCG) included patients with proven bloodstream infection (detection of a causative pathogen in blood culture) and/or clinical evidence of complicated infection (escalation of antibiotic treatment, clinical deterioration after start of antibiotics). In the second group (mild course group, MCG) we analyzed samples from patients with FN, no clinical signs for sepsis, and normalization of body temperatures within 48 h after start of antibiotic treatment. Patient characteristics are shown in Table 1.

**Table 1:** Patient characteristics

	Patients *N* = 33	Febrile episodes *N* = 47	
		MCG^a^	SCG^b^
		*N* = 31 (66.0%)*	*N* = 16 (34.0%)*
Median age at oncologic diagnosis in years (25th–75th quartile)	8.65 (3.75–14.54)	–	–
Median age at febrile episode in years (25th–75th quartile)	–	8.56 (3.20–13.42)	12.99 (6.86–16.64)
Male gender (percentage)	*N* = 17 (51.5%)	*N* = 17 (54.8%)**	*N* = 7 (43.8%)**
Hematological malignancies (percentage)	*N* = 22 (66.67)	*N* = 16 (51.6%)**	*N* = 11 (68.8%)**
Solid malignancies (percentage)	*N* = 11 (33.33)	*N* = 15 (48.4%)**	*N* = 5 (31.2%)**
Median Leucocytes ×109 day 1 (*n* = 20/14) (25th–75th quartile)	–	0.54 (0.20–1.23)	0.40 (0.20–0.75)
Median Leucocytes x109 day 4 (*n* = 25/15) (25th–75th quartile)	–	1.40 (0.70–2.95)	0.30 (0.20–0.65)
Median Leucocytes x109 day 7 (*n* = 18/13) (25th–75th quartile)	–	3.95 (2.33–6.98)	0.50 (0.30–1.75)
Median CRP (mg/dl) day 1 (*n* = 14/8) (25th–75th quartile)	–	47.30 (18.03–101.00)	87.55 (23.05–166.00)
Median CRP (mg/dl) day 4 (*n* = 10/7) (25th–75th quartile)	–	23.60 (17.30–62.78)	96.00 (93.60–226.00)
Median CRP (mg/dl) day 7 (*n* = 6/5) (25th–75th quartile)	–	13.70 (2.85–98.73)	61.10 (29.80–81.80)
Duration of Hospitalization in days (*n* = 24/9) (25th–75th quartile)	–	7 (4.5–8)	15 (9–16)

* Percent of total number. ** Percent of *N* = 31 (MCG) and *N* = 16 (SCG), respectively

^a^Mild course group

^b^Severe course group

### Clinical definitions

Fever was defined as a single body temperature (measured in ear or mouth) over 38.5 °C or two temperatures between 38.0 and 38.5 °C during a 1-h interval [24]. Neutropenia was defined as an absolute neutrophil count < 0.5 × 109/L and/or leucocyte count < 1.0 × 109/L with an expected decline.

### Ex-vivo sepsis model

Heparinized full blood samples were suspended in Roswell Park Memorial Institute (RPMI) 1640 supplemented with 1% penicillin/streptomycin, 2 mM glutamine, 1 mM pyruvate, and nonessential amino acids (Seromed Biochrome, Berlin, Germany) at a concentration of 5 × 109 leukocytes/L within 24 h after collection. The leucocyte count was measured at the time of sampling. Lipopolysaccharide (LPS, 30 ng/ml) or *Staphylococcus epidermidis* (SE; ATCC 12490; 1-10 colony forming unit/white blood cell) were added in order to stimulate cytokine production. These “stimulated samples” were transferred to cell culture flasks and incubated for 24 h with 37 °C in 5 % CO_2_. There was no red blood cell lysis performed. Afterwards, cells were separated and cell culture supernatant was stored at − 80 °C. Incubated full blood samples without stimulation with LPS or SE (“unstimulated samples”) served as controls.

### Cytokine assays

Cytokines from whole blood culture supernatants were measured using the cytometric bead array Bio-Plex Pro™ Human Cytokine 27-plex Assay and Bio-Plex® 200 system. All parameters were measured in ng/ml. We measured the following cytokines: IL-1β, IL-1ra, IL-2, IL-4, IL-5, IL-6, IL-7, IL-8, IL-9, IL-10, IL-12, IL-13, IL-15, IL-17, Eotaxin, FGF-β, G-CSF, GM-CSF, IFN-γ, IP-10, MCP-1, MIP-1α, PDGF-BB, MIP-1β, RANTES, TNF-α, and VEGF. We excluded cytokines, which were detected in less than two-thirds of the samples for final evaluation (IL-2, IL-5, IL-7, IL-12, IL-13, IL-15, and VEGF).

### Statistics

Statistical analysis was performed using SPSS 22.0 (SPSS Inc., Chicago, Il, USA). Patient characteristics were evaluated using descriptive statistical methods; variables were expressed as median and quartiles. Statistical differences were tested for paired data by using Wilcoxon-rank sum test and Friedman test. Results of the Friedman test were corrected by the Bonferroni correction. For unpaired data, we used the Mann-Whitney *U* test. In all tests, *p* values < 0.05 were considered significant. All febrile neutropenia episodes were treated as statistically independent, including repeated episodes in the same patients.

## Results

### Study cohort

In this study we included data of 47 febrile neutropenic episodes from 33 children receiving chemotherapy. In four episodes, there was a culture-proven infection (*Pseudomonas paucimobilis*, *Proteus mirabilis*, *Klebsiella pneumoniae*, *Escherichia coli*). In one of them, a *Candida albicans* infection was additionally found. No patient died during the febrile neutropenic episodes. Detailed patient characteristics are shown in Table 1.

FN was treated with Tazobactam and Piperacillin in 93.62% of cases, 57.45% of these patients received Tobramycin in addition. Three patients were treated with Clindamycin and Gentamycin (2.13%), Clarithromycin (2.13%), or Ceftazidime (2.13%). In the SCG, escalation of antibiotic treatment was performed in 15 episodes and contained Meropenem (13.33%), Teicoplanin (6.67%), Meropenem plus Teicoplanin (20.0%), Vancomycin (20.0%), Vancomycin plus Meropenem (26.67%), and Metronidazole (6.67%). The median day of antibiotic escalation was day 4 of FN.

### Cytokine expression over time

During the febrile episodes, there was a significant increase of cytokine concentration from day 1 to day 7, beginning at day 4 after LPS and/or SE stimulation. IL-8 expression significantly increased after LPS as well as after SE stimulation from day 1 to 7. IL-1β and MCP-1 expression only increased significantly after LPS stimulation (data not shown). There was no statistically significant difference between the power of LPS and SE to stimulate baseline cytokine expression. Every single expression value for all cytokines at each point of time is shown in the supplemental table 1.

### Cytokine expression by risk stratification

**Fig. 1:**
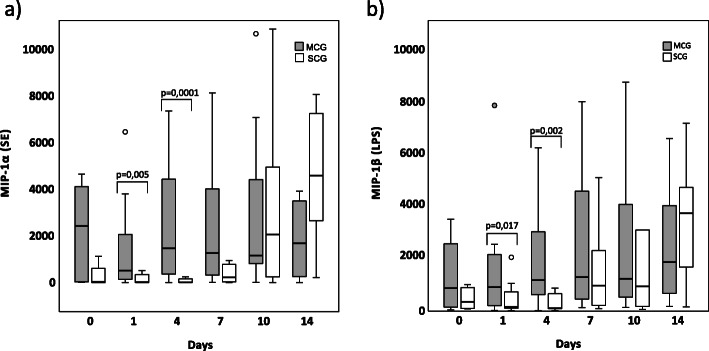
**a** MIP-1α expression after SE-stimulation and **b** MIP-1β expression after LPS-stimulation in pg/ml on days 0, 1, 4, 7, 10, and 14 during febrile neutropenia. Dark bars show expression levels in MCG patients, light bars show the same for SCG patients. Data are presented as box plots indicating median, 25%/75% quartiles and outliers with an interquartile range < 1.5. *P* values are derived from Mann-Whitney *U* test. *P* values are just calculated for days 1 and 4.

Sixteen of the 47 febrile episodes were classified as severe courses (SCG) and 31 as mild courses (MCG). According to the relevance of clinical decision-making in daily hospital routine, we focused our analysis on days 1 and 4 after onset of fever. After both, SE and LPS stimulation, IL-8, MIP-1α, MIP-1β, MCP-1, and TNF-α showed significantly lower concentration in the SCG as compared to the MCG at both time points. One representative graph is depicted (Fig. 1). IL-1β and IL-1ra, however, only showed significantly lower expression levels in the SCG after SE stimulation and IL-6 only after LPS stimulation (data not shown).

There was a marked median leucopenia at day 1 of the episodes (0.5 × 10^9^/L) and the main increase of median leukocyte count was seen between day 4 (0.85 × 10^9^/L) and day 7 (2.4 × 10^9^/L) (data not shown). As expected, median leucocyte counts in SCG increased later than in the MCG group (supplemental fig. 1).

### Pro- and anti-inflammatory ratio

**Fig. 2:**
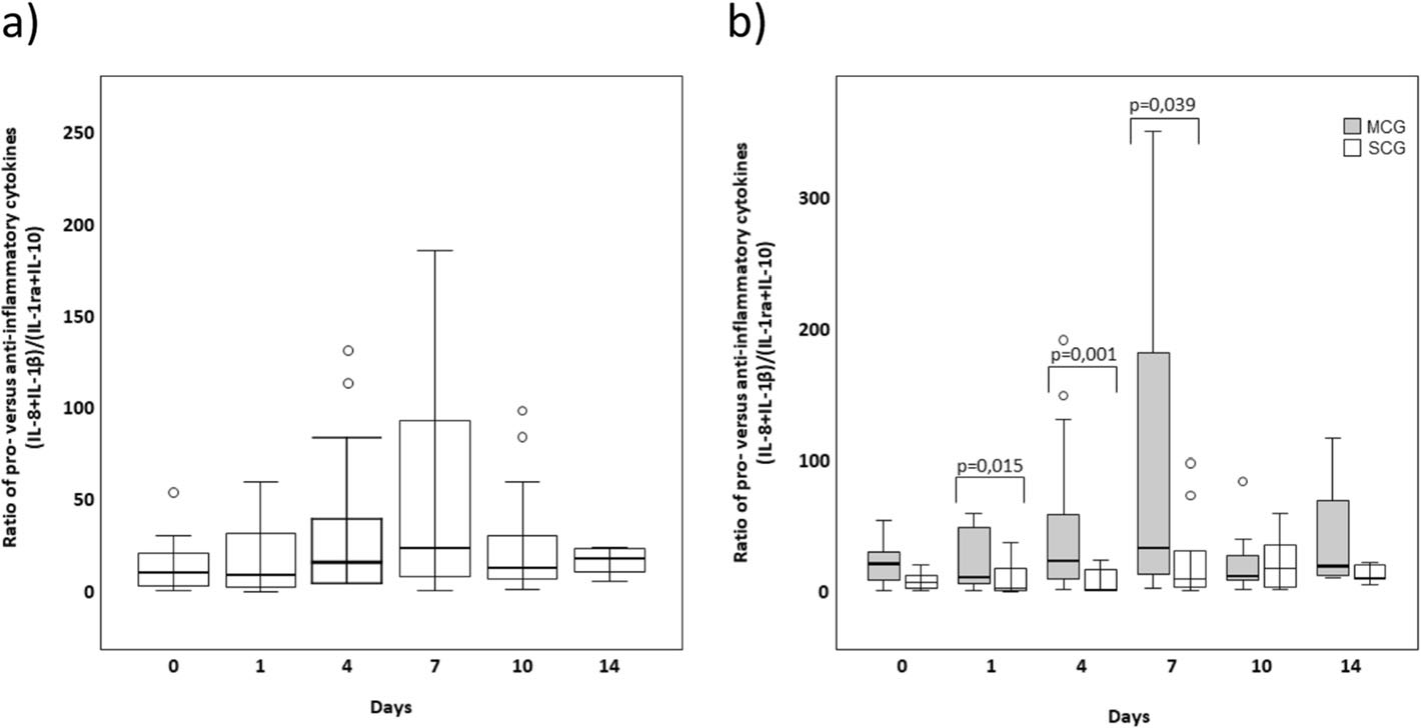
Ratio of the pro- and anti-inflammatory cytokines (IL-8 + IL-1β) / (IL-1ra + IL-10) during febrile neutropenia (**a**). Part **b** shows the same ratio differentiated for patients with mild and severe clinical courses. Data are presented as box plots indicating median, 25%/75% quartiles and outliers with an interquartile range < 1.5. *P* values are derived from Mann-Whitney *U* test (**b**).

Dysbalances in pro- and anti-inflammatory cytokines can be found in sepsis patients. Therefore, we chose pro-inflammatory cytokines which act as key actors in the regulation of infections (IL-1β plus IL-8) and divided the expression of the sum of both by anti-inflammatory cytokines (IL-1ra plus IL-10) to calculate a pro- and anti-inflammatory cytokine expression ratio. IL-1ra was chosen, since it acts as antagonist of IL-1β; IL-10 directly inhibits the inflammatory macrophages and thereby decreases the expression of pro-inflammatory cytokines. In addition, we calculated a second ratio only including the antagonists IL-1β and IL-1ra. We found that pro-inflammatory cytokines increased with time (Fig. 2a) and in parallel to leukocyte recovery (timeline of leukocyte recovery is shown in suppl. fig.). Predominance of pro-inflammatory cytokines was more pronounced in MCG than in SCG episodes (Fig. 2b). Same results were found in the ratio of four blood culture positive samples. There were no differences between both clinical risk groups concerning the IL-1β and IL-1ra ratio.

### Cytokine expression stratified according to the type of malignant disease

SCG episodes were more frequent in the group of patients with hematological malignancies (*p* = 0.028, Table 1). All other strata were equally distributed. Neither LPS nor SE stimulated samples showed consistent differences between both groups. Only IFN-γ and MCP-1 show significant lower concentrations on more than one time point (days 4 and 7) in patients with hematological diseases after LPS stimulation (data not shown).

## Discussion

Children with febrile neutropenia receive broad-spectrum antibiotic treatment, although bacterial infection cannot be proven in the majority of episodes. Clinical models to predict microbiologically defined infections have been established [15]; however, reliable biomarkers are still needed to differentiate patients with bacterial infection from those without infection.

The role of cytokines as infection markers is still subject to controversy even though they are well known for their crucial function in systemic inflammation. We investigated cytokine regulation during febrile neutropenia in childhood cancer patients and found that three cytokines (IL-8, MCP-1, and IL-1β) increase during febrile neutropenia in an ex vivo sepsis model. In addition, we found that pro-inflammatory cytokines exceed anti-inflammatory cytokines during leukocyte recovery and that low IL-8, MIP-1α, MIP-1β, MCP-1, and TNF-α expression at the beginning of the febrile episodes seem to hint at a more severe clinical course of the infection.

All cytokines indicating a severe clinical course are pro-inflammatory cytokines, secreted by macrophages, fibroblasts, endothelial cells (MCP-1, IL-8), and T cells (TNF-α) [25, 26]. IL-8 activates neutrophils, basophils, and T cells and is one of the most prominently discussed cytokines in the context of febrile neutropenia [27, 28]. IL-8, together with other markers like PCT or IL-6, was found to be a reliable diagnostic marker for sepsis or bacteremia [5, 11, 18, 29, 30] and to predict low-risk for bacterial infections [31]. However, IL-8 was also found not to be able to predict bacterial infections in febrile neutropenia in children [16, 32]. MIP-1α and MIP-1β activate monocytes, macrophages, and granulocytes but also induce production of IL-6, IL-1α, and TNF-α [31–33]. These cytokines are described in the context of sepsis as possible predictors of outcome; however, not in the context of FN [34, 35]. MCP-1 affects activation of monocytes, macrophages, and T cells and its expression is correlated with mortality in non-neutropenic sepsis patients and again, its role in context of febrile neutropenia in children has not yet been investigated [34, 36, 37]. TNF-α induces fever and shock. Its expression strongly depends on the regulation of IL-6 and IL-10 and, therefore, varies interindividually [38, 39]. Hence, its role as a marker for febrile neutropenia could not be determined yet [29, 40, 41].

Our study suggests that further prospective randomized trials are needed to evaluate cytokine profiles in ex vivo stimulation models as diagnostic markers. These markers could be an additional tool to support antibiotic stewardship predicting a severe course of febrile neutropenia. Due to the high biological complexity of the inflammasome and its stimulation by chemotherapy-induced neutropenia, it would be plausible that cytokine regulation may not be diagnosed by measurement of a single cytokine alone but a number of different cytokines and the calculation of a pro-/anti-inflammatory cytokine ratio. In our study, we found an association of three cytokines and the development of febrile neutropenia and, in addition, five potential candidates for the discrimination between a severe and a mild course of FN episodes. Other groups using unstimulated samples found different results for cytokine regulation [12, 31, 32, 42]. However, none of these groups had investigated high numbers of cytokines at the same time. In addition, none of these groups were able to calculate pro-/anti-inflammatory cytokine ratios to show that regulation of single cytokines does not represent the entirety of cytokine regulation. We demonstrated that the combined pro-/anti-inflammatory ratio is even more suitable to discriminate between severe and mild courses of FN than the antagonists IL-1ra and IL-1β.

As expected, leukocyte count increased during time of the febrile episode and this happened earlier in patients with MCG than in those with SCG. In daily routine, this parameter is not used as a predictive value for infection severity, but especially the neutrophil count is used as a parameter for termination of the antibiotic treatment [17]. Of interest, the increase of leukocyte recovery was accompanied with an increasing pro- and anti-inflammatory cytokine ratio, whereas differential regulation of single cytokines was observed earlier. This suggests that (a) basic immune regulation is present even in neutropenic patients and (b) immune recovery precedes cellular recovery. One may interpret the pro- and anti-inflammatory cytokine regulation as a hallmark of systemic inflammation and it would be interesting to know, whether sustained inflammation is associated with long-term comorbidity of the patients. However, this would be subject to future studies.

In this single-center observational study, we focused on the expression pattern of cytokines during febrile neutropenia in an ex vivo sepsis model. We chose this for its benefit of a minor dependence on limiting external (e.g., day time) and internal (patients intrinsic stress, regulation of hormones) potential influence factors. In addition, we aimed at achieving high levels of cytokines in order to facilitate the discrimination of cytokine patterns. The main strength of our study is its contribution to define early biomarkers for discrimination of severe and mild clinical courses of FN, even though they need to be validated by prospective studies. Nevertheless, they might be able to support early clinical decision making. In the context of chronically sick children and the burden of increasing antibiotic resistances, shortening of antibiotic treatment of even a few days could already have a major impact. However, this study has several limitations. First, the long incubation time needed for ex vivo stimulation as well as the complex protocol limits the use of our model for routine clinical diagnostics. By a recent multivariable meta-analysis including a variety of clinical and disease-related parameters as well as laboratory findings, Phillips et al. defined a model of determining an individual’s chance of microbiologically defined infection during febrile neutropenia episode [15]. This model finally consists of six components: malignancy type, temperature, clinical description of being severely unwell, hemoglobin level, total white blood cell count, and absolute monocyte count. This supports the findings of our study, showing that rather a combination of different inflammation/infection parameters might serve as a reliable model to discriminate bacterial infection from non-infectious episodes. A second limitation of our study might be the retrospective division of our cohort into the two groups of MCG and SCG, since this did not follow prospectively defined criteria. Antibiotic treatment of these groups was not identical, since escalation of antibiotics defined the SCG and antibiotic escalation itself might have influenced the expression of cytokines. In addition, supportive therapies such as Amphotericin B or Fluconazole were used more often in SCG than in MCG.

In conclusion, we propose that the regulation of cytokine levels as measured in a small panel of different cytokines could attribute to risk stratification of febrile neutropenia. Future prospective studies are needed to evaluate this distinct FN signature as a diagnostic biomarker and a prognostic tool for individualized strategies.

## Supplementary information

**Additional file 1: **Supplemental Figure 1: Median leukocyte count (×10^9^/L) of MCG and SCG patients on days 0, 1, 4, 7, 10, and 14 of the episodes. Data are presented as box plots indicating median, 25%/75% quartiles and outliers with an interquartile range < 1.5. *P* values are derived from Mann-Whitney *U* test.

**Additional file 2:.** Supplemental Table 1.

## Data Availability

The datasets used and analyzed during the current study are available from the corresponding author on reasonable request.

## Abbreviations

CRP: C-reactive protein
FGF-β: Fibroblast growth factor-β
G-CSF: Granulocyte colony-stimulating factor
GM-CSF: Granulocyte-macrophage colony-stimulating factor
IFN-γ: Interferon-γ
IL: Interleukin
IP-10: Interferon gamma-induced protein-10
LPS: Lipopolysaccharide
MCG: Mild course group
MIP-1α: Macrophage-inflammatory protein-1α
MIP-1β: Macrophage-inflammatory protein-1β
MCP-1: Monocyte chemotactic protein-1
PCT: Procalcitonin
PDGF-BB: Platelet-derived growth factor-BB
RANTES: Regulated upon activation normal T cell expressed and secreted
RPMI: Roswell Park Memorial Institute
SCG: Severe course group
SE: *Staphylococcus epidermidis*
TNF-α: Tumor necrosis factor-α
VEGF: Vascular endothelial growth factor
