# Supplementary figures and images for: Pro-inflammatory cytokine ratios determine the clinical course of febrile neutropenia in children receiving chemotherapy
Source: Mol Cell Pediatr. 2020 Jun 9;7:5. doi: 10.1186/s40348-020-00097-2 (PMC7283414; doi:10.1186/s40348-020-00097-2)

## Slide 1
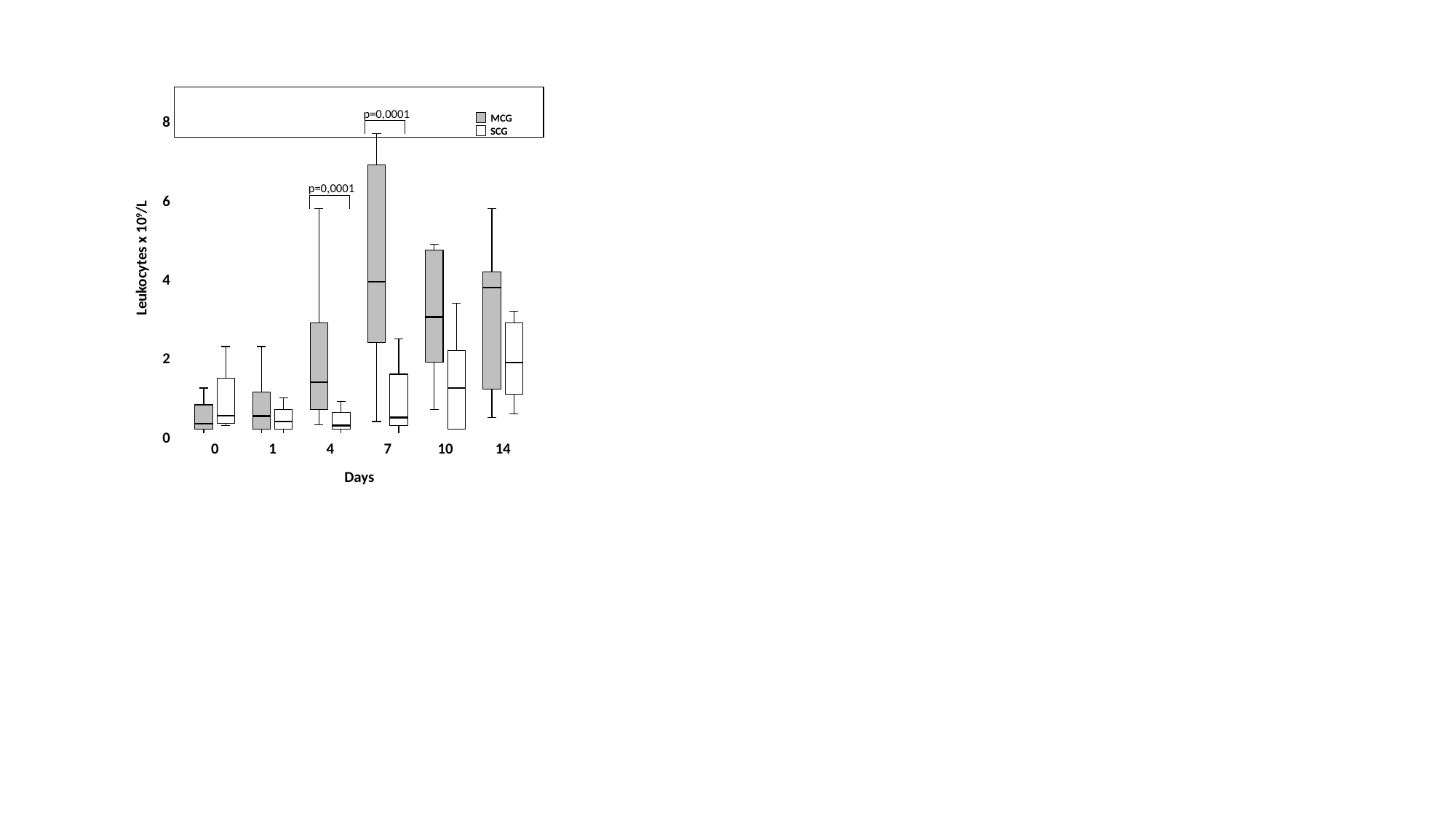

MCG SCG
p=0,0001
8
p=0,0001
Leukocytes x 109/L
6
4
2
0
0	1	4	7	10	14
Days

Supplement: Supplementary file 1 — Additional file 1: Supplemental Figure 1: Median leukocyte count (×109/L) of MCG and SCG patients on days 0, 1, 4, 7, 10, and 14 of the episodes. Data are presented as box plots indicating median, 25%/75% quartiles and outliers with an interquartile range < 1.5. P values are derived from Mann-Whitney U test. [file 40348_2020_97_MOESM1_ESM.pptx]
