# Supplementary material for: Pro-inflammatory cytokine ratios determine the clinical course of febrile neutropenia in children receiving chemotherapy
Source: Mol Cell Pediatr. 2020 Jun 9;7:5. doi: 10.1186/s40348-020-00097-2 (PMC7283414; doi:10.1186/s40348-020-00097-2)
Supplement: Supplementary file 2 — Additional file 2:. Supplemental Table 1. [file 40348_2020_97_MOESM2_ESM.docx]

**Supplemental table**

| **Cytokine**  **LPS** | **Day 0**  **x10^-15^/L, N=18** | **Day 1**  **x10^-15^/L, N=45** | **Day 4**  **x10^-15^/L, N=47** | **Day 7**  **x10^-15^/L, N=37** | **Day 10**  **x10^-15^/L, N=26** | **Day 14**  **x10^-15^/L, N=15** |
| --- | --- | --- | --- | --- | --- | --- |
| IL-1β  (25^th^ – 75^th^ quartile) | 18.08  (0.03 - 369.68) | 47.41  (1.87 - 571.07) | 324.6  (5.87 - 3,361.60) | 690.5  (23.84 - 3,420.21) | 1,035.46  (93.45 - 5,567.58) | 1,232.39  (208.75 - 7,168.13) |
| IL-1ra  (25^th^ – 75^th^ quartile) | 240.59  (0.00 - 513.42) | 167.93  (36.77 - 517.73) | 437.86  (97.67 - 1,471.79) | 396.03  (103.21 - 1,675.57) | 1,195.88  (309.24 - 4,206.80) | 1,689.19  (661.68 - 2,796.46) |
| IL-2  (25^th^ – 75^th^ quartile) | 0.55  (0.00 - 19.74) | 2.89  (0.00 - 17.98) | 2.55  (0.00 - 9.69) | 0.00  (0.00 - 8.97) | 0.00  (0.00 - 11.08) | 0.00  (0.00 - 0.00) |
| IL-4  (25^th^ – 75^th^ quartile) | 2.88  (0.00 - 12.77) | 3.07  (1.01 - 16.48) | 3.75  (2.11 - 17.18) | 3.47  (1.05 - 14.57) | 3.71  (1.76 - 21.04) | 4.52  (1.23 - 23.13) |
| IL-5  (25^th^ – 75^th^ quartile) | 22.04  (0.00 - 83.41) | 0.00  (0.00 - 43.77) | 0.00  (0.00 - 47.36) | 0.00  (0.00 - 38.53) | 0.00  (0.00 - 30.11) | 0.00  (0.00 - 30.40) |
| IL-6  (25^th^ – 75^th^ quartile) | 18.75  (3.63 - 137.26) | 23.53  (3.45 - 158.05) | 21.61  (7.19 - 156.86) | 10.31  (1.19 - 130.79) | 49.28  (11.32 - 159.71) | 36.06  (0.00 - 141.18) |
| IL-7  (25^th^ – 75^th^ quartile) | 0.00  (0.00 - 28.67) | 3.08  (0.00 - 34.94) | 0.00  (0.00 - 18.85) | 0.00  (0.00 - 22.90) | 0.00  (0.00 - 4.55) | 0.00  (0.00 - 59.70) |
| IL-8  (25^th^ – 75^th^ quartile) | 1,770.82  (113.68 - 9,058.08) | 1,163.53  (134.49 - 9,743.17) | 9,565.22  (614.97 - 2,2513.26) | 10,993.5  (5,353.64 - 28,848.96) | 16,778.25  (8,938.24 - 27,809.58) | 12,654.40  (8,332.40 - 99,216.27) |
| IL-9  (25^th^ – 75^th^ quartile) | 93.25  (47.04 - 191.45) | 117.99  (63.97 - 340.94) | 107.75  (53.49 - 440.47) | 144.7  (63.16 - 574.65) | 86.13  (50.43 - 808.76) | 80.76  (56.07 - 748.40) |
| IL-10  (25^th^ – 75^th^ quartile) | 2.40  (1.85 - 23.09) | 2.23  (0.12 - 26.77) | 2.79  (1.13 - 14.67) | 2.90  (0.30 - 16.21) | 3.29  (1.42 - 8.93) | 3.93  (2.40 - 27.84) |
| IL-12  (25^th^ – 75^th^ quartile) | 0.00  (0.00 - 34.09) | 2.47  (0.00 - 79.94) | 1.53  (0.00 - 31.58) | 0.00  (0.00 - 57.14) | 0.00  (0.00 - 48.49) | 0.00  (0.00 - 66.20) |
| IL-13  (25^th^ – 75^th^ quartile) | 0.00  (0.00 - 17.21) | 4.29  (0.00 - 52.14) | 0.00  (0.00 - 16.94) | 0.00  (0.00 - 2.67) | 0.00  (0.00 - 8.38) | 0.00  (0.00 - 16.94) |
| IL-15  (25^th^ – 75^th^ quartile) | 610.22  (145.21 - 755.51) | 152.52  (0.00 - 727.51) | 275.23  (0.00 - 695.89) | 341.82  (0.00 - 695.89) | 688.72  (0.00 - 1,033.13) | 667.47  (0.00 - 1,089.05) |
| IL-17  (25^th^ – 75^th^ quartile) | 62.75  (2.31 - 261.44) | 86.56  (16.57 - 352.04) | 96.42  (47.48 - 327.73) | 86.56  (31.46 - 430.65) | 115.67  (41.65 - 548.91) | 99.61  (49.70 - 543.91) |
| Eotaxin  (25^th^ – 75^th^ quartile) | 13.02  (0.00 - 35.98) | 11.57  (6.87 - 53.45) | 14.41  (5.90 - 51.31) | 11.4  (3.38 - 55.73) | 6.80  (1.66 - 63.03) | 9.20  (1.68 - 69.83) |
| FGF-β  (25^th^ – 75^th^ quartile) | 130.99  (27.83 - 216.04) | 107.94  (54.99 - 234.10) | 149.87  (83.68 - 294.68) | 137.45  (53.52 - 334.20) | 132.57  (72.81 - 384.08) | 136.45  (68.54 - 563.05) |
| G-CSF  (25^th^ – 75^th^ quartile) | 429.55  (239.64 - 606.61) | 277.08  (87.47 - 568.94) | 481.31  (98.65 - 689.70) | 392.21  (77.76 - 811.15) | 500.37  (217.47 - 1,335.00) | 951.95  (280.83 - 1,529.23) |
| GM-CSF  (25^th^ – 75^th^ quartile) | 12.24  (0.00 - 1,170.02) | 515.4  (0.00 - 1,147.30) | 424.46  (0.00 - 1,176.01) | 427.51  (3.57 - 1,095.96) | 15.03  (0.00 - 1,265.42) | 18.05  (0.00 - 1,247.51) |
| IFN-γ  (25^th^ – 75^th^ quartile) | 28.44  (13.03 - 578.64) | 152.31  (14.07 - 788.62) | 240.47  (16.48 - 644.63) | 176.54  (15.11 - 834.31) | 44.97  (10.98 - 881.94) | 42.55  (10.87 - 1,224.99) |
| IP-10  (25^th^ – 75^th^ quartile) | 17.67  (0.00 - 123.54) | 29.34  (19.51 - 100.19) | 33.5  (16.40 - 86.16) | 29.12  (12.65 - 73.92) | 40.84  (13.39 - 78.63) | 39.34  (0.00 - 85.90) |
| MCP-1  (25^th^ – 75^th^ quartile) | 291.32  (39.45 - 791.62) | 425.25  (60.51 - 806.01) | 737.45  (127.81 - 1,699.70) | 651.67  (329.92 - 1,693.51) | 1,119.00  (345.53 - 1,851.46) | 1,312.45  (313.07 - 1,850.56) |
| MIP-1α  (25^th^ – 75^th^ quartile) | 45.13  (5.63 - 2,667.82) | 123.78  (11.51 - 1,809.39) | 745.22  (18.18 - 4,809.68) | 852.65  (72.75 - 4,085.82) | 2,186.79  (523.98 - 4,247.37) | 3,760.63  (1,030.42 - 5,108.66) |
| PDGF-BB  (25^th^ – 75^th^ quartile) | 1,635.20  (643.18 - 5,176.49) | 1,394.48  (569.92 - 4,464.02) | 2,841.88  (570.79 - 5,745.45) | 1,701.14  (561.64 - 8,696.76) | 5,879.84  (764.64 - 14,087.94) | 7,778.25  (2,164.87 - 16,461.62) |
| MIP-1β  (25^th^ – 75^th^ quartile) | 528.09  (107.27 - 1,896.43) | 434.42  (132.27 - 1,818.57) | 853.02  (159.57 - 2,953.49) | 1,111.66  (225.45 - 3,602.12) | 1,167.21  (491.79 - 3,717.51) | 2,578.66  (1,022.35 - 4,447.04) |
| RANTES  (25^th^ – 75^th^ quartile) | 13,713.77  (6,451.93 - 39,522.29) | 17,939.26  (6,213.19 - 44,604.34) | 8,181.74  (3,457.70 - 33,086.71) | 9,408.26  (3,758.87 - 28,624.57) | 13,703.33  (5,283.68 - 39,432.00) | 24,915.18  (5,695.97 - 133,266.76) |
| TNF-α  (25^th^ – 75^th^ quartile) | 89.64  (12.46 - 547.88) | 166.2  (37.45 - 557.48) | 249.48  (93.19 - 789.49) | 291.07  (95.25 - 875.42) | 662.12  (195.51 - 1,151.57) | 689.63  (517.89 - 1,403.57) |
| VEGF  (25^th^ – 75^th^ quartile) | 16.60  (0.00 - 220.63) | 40.94  (0.00 - 179.77) | 52.77  (0.00 - 336.42) | 75.3  (0.00 - 510.21) | 213.36  (0.00 - 348.54) | 560.55  (0.00 - 871.58) |

| **Cytokine**  **SE** | **Day 0**  **x10^-15^/L, N=17** | **Day 1**  **x10^-15^/L, N=46** | **Day 4**  **x10^-15^/L, N=46** | **Day 7**  **x10^-15^/L, N=35** | **Day 10**  **x10^-15^/L, N=25** | **Day 14**  **x10^-15^/L, N=15** |
| --- | --- | --- | --- | --- | --- | --- |
| IL-1β  (25^th^ – 75^th^ quartile) | 13.98  (0.00 - 1,120.16) | 86.48  (8.68 - 593.07) | 239.32  (6.99 - 1,339.57) | 335.00  (17.33 - 2,558.56) | 944.15  (38.16 - 3,901.33) | 1,219.48  (42.38 - 3,858.56) |
| IL-1ra  (25^th^ – 75^th^ quartile) | 203.95  (0.00 - 801.97) | 329.17  (62.12 - 704.55) | 543.49  (125.99 - 1,193.30) | 456.70  (145.26 - 1,144.30) | 1,509.23  (368.57 - 4,685.03) | 2,616.42  (673.71 - 4,702.53) |
| IL-2  (25^th^ – 75^th^ quartile) | 0.99  (0.00 - 18.83) | 4.55  (0.00 - 15.70) | 0.00  (0.00 - 11.51) | 0.00  (0.00 - 10.50) | 0.00  (0.00 - 14.86) | 0.00  (0.00 - 8.47) |
| IL-4  (25^th^ – 75^th^ quartile) | 3.63  (0.00 - 14.20) | 3.06  (1.39 - 16.92) | 3.93  (1.03 - 14.24) | 3.32  (0.24 - 11.70) | 3.25  (1.47 - 22.26) | 4.18  (1.74 - 12.09) |
| IL-5  (25^th^ – 75^th^ quartile) | 26.59  (0.00 - 94.97) | 7.91  0.00 - 55.35) | 0.00  (0.00 - 45.75) | 0.00  (0.00 - 20.44) | 0.00  (0.00 - 49.55) | 0.00  (0.00 - 27.37) |
| IL-6  (25^th^ – 75^th^ quartile) | 8.61  (4.04 - 528.14) | 37.39  (3.55 - 200.64) | 10.81  (1.53 - 93.25) | 4.89  (0.05 - 127.02) | 27.60  (1.14 - 133.40) | 8.20  (6.08 - 127.20) |
| IL-7  (25^th^ – 75^th^ quartile) | 0.00  (0.00 - 44.08) | 6.93  (0.00 - 35.02) | 4.73  (0.00 - 29.59) | 2.05  (0.00 - 21.67) | 0.00  (0.00 - 23.44) | 0.00  (0.00 - 42.61) |
| IL-8  (25^th^ – 75^th^ quartile) | 935.21  (240.00 - 15,861.30) | 2,118.66  (346.79 - 8,319.51) | 8,473.54  (606.42 - 15,504.92) | 9,103.59  (3,136.58 - 27,413.81) | 13,936.20  (4,490.97 - 33,128.14) | 13,760.43  (4,679.22 - 37,712.53) |
| IL-9  (25^th^ – 75^th^ quartile) | 87.05  (46.78 - 293.38) | 127.04  (77.85 - 338.09) | 121.52  (55.57 - 535.00) | 160.37  (67.34 - 543.86) | 95.35  (52.71 - 912.12) | 98.74  (66.70 - 698.99) |
| IL-10  (25^th^ – 75^th^ quartile) | 2.23  (1.22 - 21.42) | 2.07  (0.00 - 32.29) | 2.40  (0.64 - 18.71) | 2.40  (0.00 - 29.84) | 2.90  (0.75 - 20.23) | 3.15  (1.09 - 25.79) |
| IL-12  (25^th^ – 75^th^ quartile) | 0.00  (0.00 - 53.83) | 4.33  (0.00 - 73.95) | 2.95  (0.00 - 42.19) | 5.28  (0.00 - 57.53) | 0.00  (0.00 - 69.01) | 0.00  (0.00 - 80.04) |
| IL-13  (25^th^ – 75^th^ quartile) | 2.94  (0.00 - 31.67) | 6.15  (0.00 - 52.91) | 0.00  (0.00 - 9.28) | 0.00  (0.00 - 7.35) | 0.00  (0.00 - 25.82) | 0.00  (0.00 - 16.94) |
| IL-15  (25^th^ – 75^th^ quartile) | 519.60  (0.00 - 745.61) | 209.82  (0.00 - 663.47) | 326.91  (0.00 - 685.95) | 256.85  (0.00 - 678.82) | 338.04  (0.00 - 880.61) | 503.67  (0.00 - 714.95) |
| IL-17  (25^th^ – 75^th^ quartile) | 79.66  (2.31 - 335.92) | 84.41  (25.84 - 345.00) | 81.52  (39.66 - 311.81) | 105.61  (23.54 - 330.07) | 104.66  (20.17 - 759.50) | 115.17  (37.43 - 416.84) |
| Eotaxin  (25^th^ – 75^th^ quartile) | 10.57  (1.32 - 45.44) | 13.14  (6.48 - 53.56) | 14.51  (6.61 - 54.65) | 12.35  (3.48 - 47.98) | 12.35  (1.77 - 77.12) | 7.99  (1.23 - 62.04) |
| FGF-β  (25^th^ – 75^th^ quartile) | 110.03  (26.99 - 219.43) | 112.52  (69.13 - 216.21) | 138.65  (73.69 - 260.74) | 117.04  (52.52 - 254.42) | 187.58  (63.25 - 367.01) | 122.26  (70.18 - 456.39) |
| G-CSF  (25^th^ – 75^th^ quartile) | 437.08  (134.32 - 542.92) | 315.12  (142.26 - 479.13) | 246.16  (79.44 - 560.85) | 250.13  (77.87 - 607.03) | 427.98  (240.22 - 917.90) | 800.54  (158.43 - 1,261.73) |
| GM-CSF  (25^th^ – 75^th^ quartile) | 5.96  (0.00 - 1,115.21) | 497.94  (0.00 - 1,152.19) | 484.60  (0.00 - 1,246.49) | 448.73  (0.00 - 1,111.40) | 15.71  (0.00 - 1,256.10) | 12.01  (0.00 - 1,129.25) |
| IFN-γ  (25^th^ – 75^th^ quartile) | 48.12  (11.80 - 767.84) | 199.07  (11.31 - 903.52) | 164.43  (20.01 - 744.91) | 105.54  (14.79 - 911.89) | 82.37  (10.99 - 975.29) | 83.77  (22.52 - 991.10) |
| IP-10  (25^th^ – 75^th^ quartile) | 19.34  (6.32 - 100.19) | 52.07  (18.80 - 121.47) | 34.57  (17.07 - 87.94) | 35.48  (16.55 - 85.90) | 45.19  (18.56 - 101.86) | 48.75  (9.61 - 157.35) |
| MCP-1  (25^th^ – 75^th^ quartile) | 118.81  (54.60 - 1,572.17) | 469.68  (85.38 - 959.60) | 679.64  (120.88 - 1,825.79) | 1,113.61  (520.58 - 1,905.19) | 1,560.57  (300.64 - 2,116.27) | 1,587.32  (599.18 - 1,948.33) |
| MIP-1α  (25^th^ – 75^th^ quartile) | 64.20  (7.25 - 3,375.41) | 265.86  (47.75 - 1,190.85) | 630.09  (43.50 - 3,857.39) | 540.86  (105.48 - 3,928.98) | 1,688.94  (369.93 - 4,895.47) | 2,780.80  (460.47 - 6,774.14) |
| PDGF-BB  (25^th^ – 75^th^ quartile) | 793.24  (434.10 - 1,887.71) | 1,246.85  (547.67 - 3,010.54) | 2,269.68  (813.55 - 5,826.67) | 3,027.76  (473.71 - 9,518.45) | 4,810.69  (1,820.80 - 14,583.46) | 4,656.43  (2,926.13 - 25,119.56) |
| MIP-1β  (25^th^ – 75^th^ quartile) | 604.72  (105.27 - 2,837.35) | 651.60  (199.87 - 1,716.79) | 1,040.88  (234.15 - 3,079.47) | 1,148.68  (289.95 - 4,156.01) | 1,833.02  (729.58 - 8,429.28) | 2,316.77  (881.83 - 5,310.36) |
| RANTES  (25^th^ – 75^th^ quartile) | 10,695.17  (5,450.62 - 23,323.63) | 11,242.31  (6,233.05 - 99,771.40) | 11,157.31  (2,900.00 - 40,341.31) | 9,325.69  (4,915.49 - 49,747.80) | 15,202.07  (8,223.49 - 36,451.75) | 19,244.71  (5,447.49 - 97,552.92) |
| TNF-α  (25^th^ – 75^th^ quartile) | 52.36  (10.46 - 696.11) | 250.53  (60.59 - 506.38) | 256.55  (77.67 - 821.20) | 224.63  (71.37 - 810.28) | 543.86  (124.91 - 1,061.55) | 596.48  (255.96 - 1,071.56) |
| VEGF  (25^th^ – 75^th^ quartile) | 26.73  (0.00 - 138.29) | 39.39  (0.00 - 154.52) | 71.92  (0.00 - 253.51) | 106.89  (0.00 - 526.54) | 178.56  (0.00 - 404.85) | 457.18  (0.00 - 1,066.27) |

Median values of all cytokines measured with SE and LPS Stimulation with 25^th^ and75^th^ quartile.
